# Tip-in underwater endoscopic mucosal resection for a residual lower rectal lesion extending to the dentate line

**DOI:** 10.1055/a-2215-2642

**Published:** 2023-12-13

**Authors:** Taishi Okumura, Kenichiro Imai, Kinichi Hotta, Sayo Ito, Yoshihiro Kishida, Kazunori Takada, Hiroyuki Ono

**Affiliations:** 1 38471 Division of Endoscopy, Shizuoka Cancer Center, Shizuoka, Japan

Endoscopic resection of lower rectal lesions close to the dentate line remains challenging because the narrow lumen in proximity to the anal sphincter results in poor visualization of the lesion 1 . Furthermore, submucosal scarring after previously attempted endoscopic removal significantly reduces the efficacy of submucosal injection. Underwater endoscopic mucosal resection (UEMR) can be effective for poorly visualized polyps, such as orifice- or diverticular-related polyps, or for scarred residual polyps 2 3 4 . Nevertheless, suboptimal visualization of the proximal margins in the deflated lumen hampers effective snaring. The tip-in maneuver ensures proximal margin visualization, achieving improved en bloc resection 5 . Here, we present a case of successful en bloc resection of a residual anorectal adenoma using tip-in UEMR ( Fig. 1 ).

**Fig. 1:**
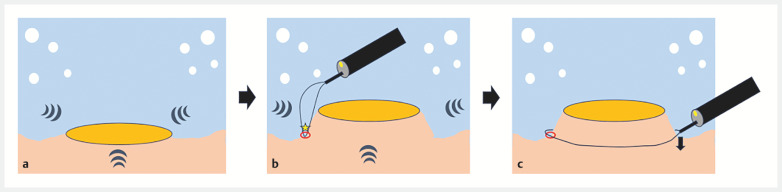
Schema of tip-in underwater endoscopic mucosal resection without injection. **a** The “floating effect” by water immersion. **b** A spot-shaped mucosal incision was created at the oral normal mucosa. Keeping the snare tip within the mucosal defect, the snare was opened. **c** By using down angulation and pushing the snare sheath down, the resection line could be controlled below the lesion.

A 79-year-old man underwent cold snare polypectomy for an 8-mm protruding anorectal polyp 3 years previously ( Fig. 2 ). Surveillance colonoscopy revealed an 8-mm residual lesion in the anorectum ( Fig. 3 ). As the diagnosis was residual adenoma, UEMR was attempted; however, snare capture was not assured because of poor visualization. To ensure proximal margin capture, a spot-shaped mucosal incision was made at the proximal side of the lesion with a snare tip using a cut current. A 15-mm rounded snare was positioned to keep the snare tip within the mucosal defect and then closed around the lesion ( Fig. 4 , Video 1 ). En bloc resection was performed without perforation. Histopathological examination revealed low grade adenoma with submucosal fibrosis ( Fig. 5 ).

**Fig. 2:**
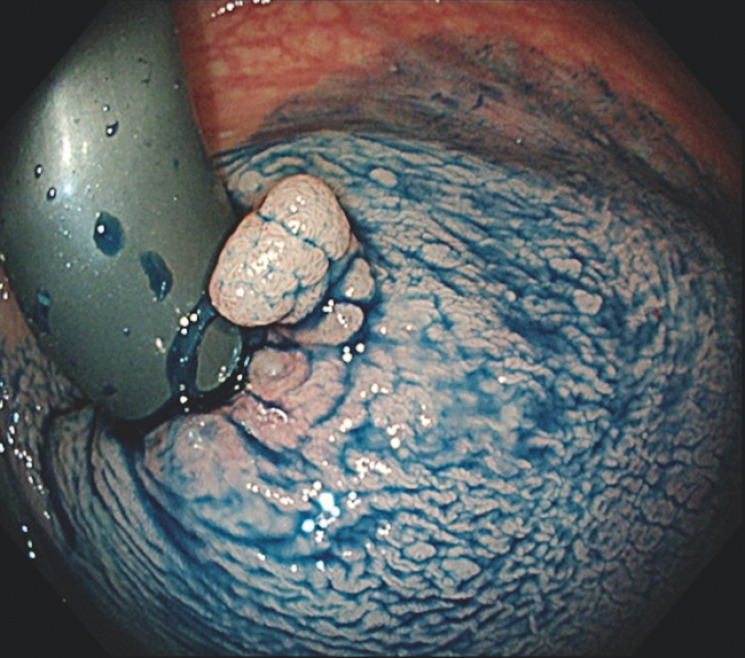
Endoscopic image showing an 8-mm protruded polyp in the lower rectum extending to the dentate line. Cold snare polypectomy was conducted 3 years previously.

**Fig. 3:**
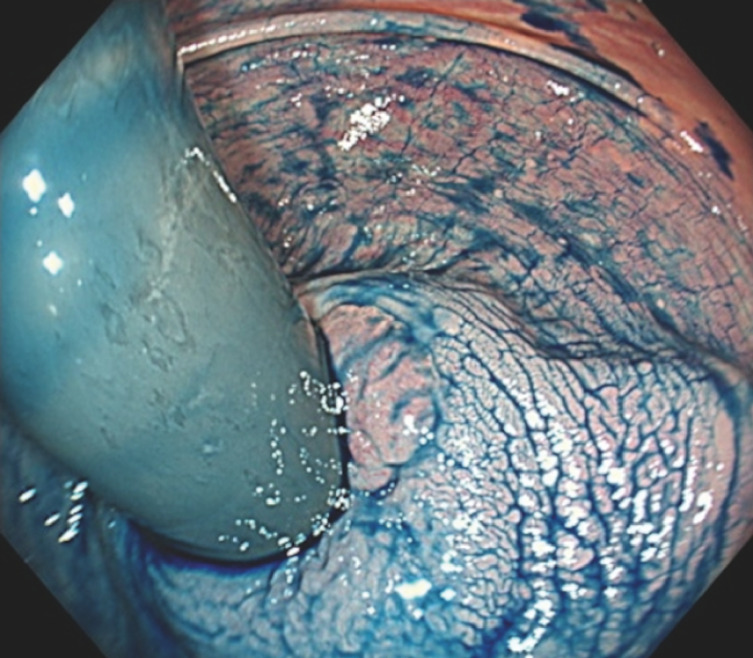
Endoscopic image during surveillance colonoscopy, showing an 8-mm residual lesion with fibrosis.

**Fig. 4:**
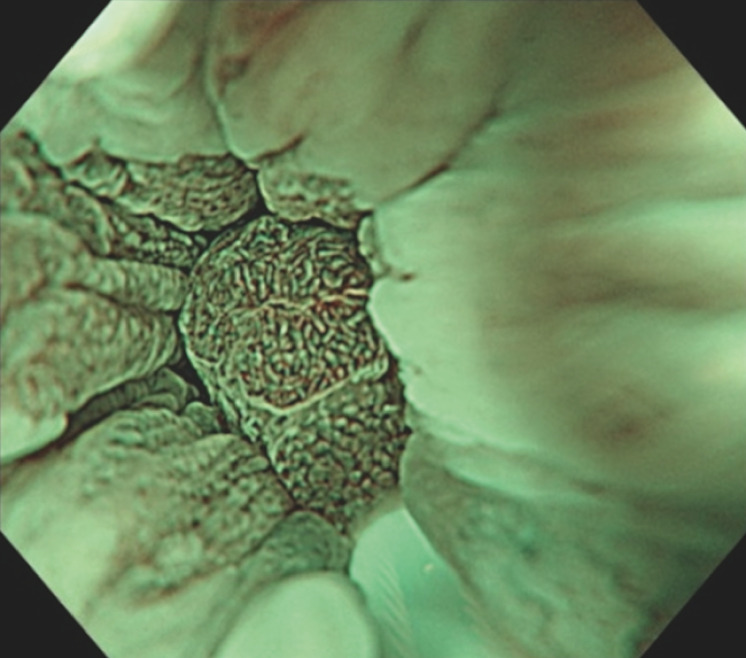
The lesion was located in the right wall. Placing the patient in the right lateral recumbent position allowed the water to pool and maximize the “floating effect.” En bloc resection was achieved.

Tip-in underwater endoscopic mucosal resection without injection as a salvage technique for a residual lower rectal lesion extending into the dentate line.Video 1

**Fig. 5:**
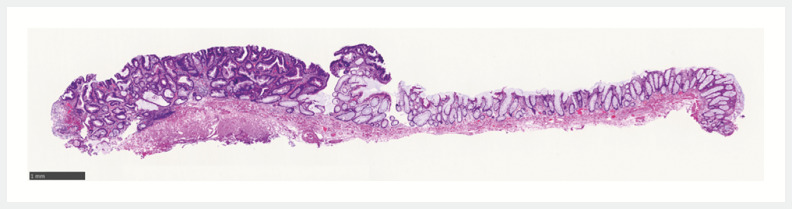
Histopathological examination showed low grade dysplasia and severe fibrosis of the submucosa.

In this case, tip-in UEMR provided several advantages. First, eliminating submucosal injections could avoid poor visualization caused by a submucosal bleb. Second, the floating effect underwater could help to snare the scarred lesion successfully. Third, the tip-in maneuver ensured proximal margin capture even in the narrow anal canal. This case suggests that tip-in UEMR is a simple and efficient technique for treating scarred anorectal adenomas.

Endoscopy_UCTN_Code_TTT_1AQ_2AD

## Notes

Endoscopy E-Videos https://eref.thieme.de/e-videos
*E-Videos* is an open access online section of the journal *Endoscopy* , reporting on interesting cases and new techniques in gastroenterological endoscopy. All papers include a high-quality video and are published with a Creative Commons CC-BY license. Endoscopy E-Videos qualify for HINARI discounts and waivers and eligibility is automatically checked during the submission process. We grant 100% waivers to articles whose corresponding authors are based in Group A countries and 50% waivers to those who are based in Group B countries as classified by Research4Life (see: https://www.research4life.org/access/eligibility/ ). This section has its own submission website at https://mc.manuscriptcentral.com/e-videos .
